# Supplementary material for: The Diagnostic Yield of Cerebrospinal Fluid Analysis for the Diagnosis of Primary Central Nervous System Lymphoma: A Systematic Review
Source: Cancers (Basel). 2025 Jul 15;17(14):2352. doi: 10.3390/cancers17142352 (PMC12293505; doi:10.3390/cancers17142352)
Supplement: Supplementary file 1 [file cancers-17-02352-s001.zip › Figure S5. A meta-logistic regression expressing the outcome of a PCNSL diagnosis using positive CSF results as a probability percentage over the period of the study..pdf]

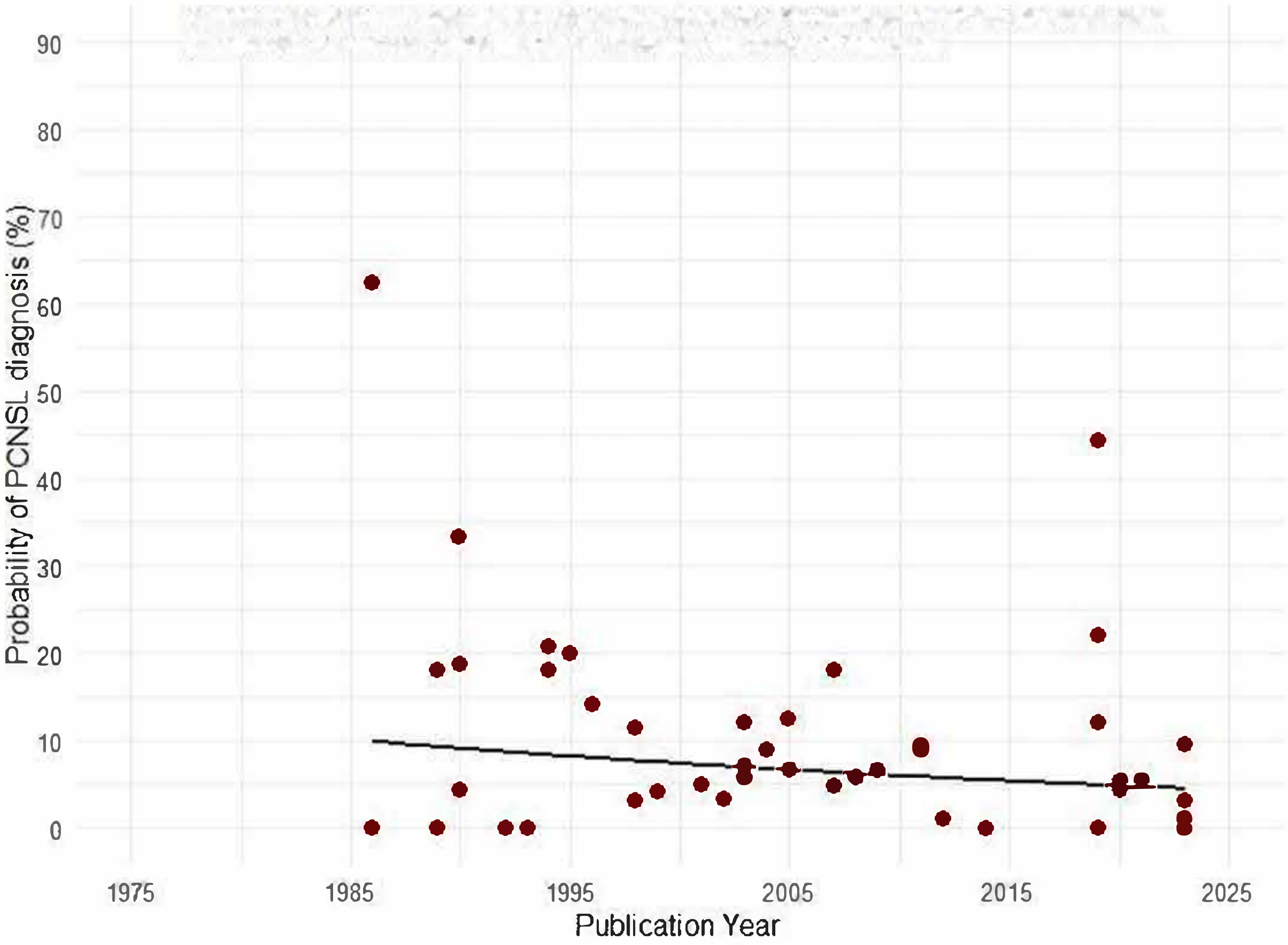

Figure S5. A meta-logistic regression expressing the outcome of a PCNSL diagnosis using positive CSF results as a probability percentage over the period of the study.
