# Supplementary material for: The Diagnostic Yield of Cerebrospinal Fluid Analysis for the Diagnosis of Primary Central Nervous System Lymphoma: A Systematic Review
Source: Cancers (Basel). 2025 Jul 15;17(14):2352. doi: 10.3390/cancers17142352 (PMC12293505; doi:10.3390/cancers17142352)
Supplement: Supplementary file 1 [file cancers-17-02352-s001.zip › Table S1. Complete list of search terms used in each database..pdf]

Table S1. Complete list of search terms used in each database.

Database	Search terms
PubMed	("PCNSL"[ti] OR ("diffuse large B-cell lymphoma*"[ti] AND ("Central Nervous System"[mesh] OR "Central Nervous System"[tiab] OR "CNS"[tiab] OR "Cerebrospinal Axis"[tiab] OR "Cerebrospinal Axes"[tiab] OR "brain"[tiab] OR "brains"[tiab] OR "cerebral"[tiab] OR "cerebro"[tiab])) OR "DLBCL"[ti] OR (("Lymphoma"[majr] OR "Lymphoma"[ti] OR "Lymphomas"[ti] OR Hodgkin*[ti]) AND ("Central Nervous System"[majr] OR "Central Nervous System"[ti] OR "CNS"[ti] OR "Cerebrospinal Axis"[ti] OR "Cerebrospinal Axes"[ti] OR "brain"[ti] OR "brains"[ti] OR "cerebral"[ti] OR "cerebro"[ti] OR "intracranial*"[ti]))) AND ("Cerebrospinal Fluid"[Mesh] OR "Cerebrospinal Fluid"[tiab] OR "Cerebrospinal Fluids"[tiab] OR "Cerebro spinal Fluid"[tiab] OR "Cerebro spinal Fluids"[tiab] OR "CSF"[ti] OR "Myeloid Differentiation Factor 88"[Mesh] OR "Myeloid Differentiation Factor 88"[tiab] OR "MyD88"[tiab] OR "CD79b"[tiab] OR "CD79 Antigens"[Mesh] OR "CD79"[tiab] OR "Chemokine CXCL13"[Mesh] OR "CXCL13"[tiab] OR "CXCL-13"[tiab] OR "beta 2-Microglobulin"[Mesh] OR "beta-2 microglobulin"[tiab] OR "beta2 microglobulin"[tiab] OR "b2 microglobulin"[tiab] OR "b2M"[tiab] OR "Neopterin"[Mesh] OR "Neopterin"[tiab] OR "neopterine"[tiab] OR "Drug Therapy"[Mesh:NoExp] OR "drug therap*"[tiab] OR "chemotherap*"[tiab] OR "Chemotherapy, Adjuvant"[Mesh] OR "Induction Chemotherapy"[Mesh] OR "Chemoradiotherapy"[Mesh] OR "chemoradiotherap*"[tiab] OR "Antineoplastic Agents"[Mesh] OR "Antineoplastic Agents" [Pharmacological Action] OR "Methotrexate"[Mesh] OR "methotrexate"[tiab] OR "MTX"[ti] OR "Stem Cell Transplantation"[Mesh] OR "Stem Cell Transplantation*"[tiab]) NOT ("animals"[mesh] NOT "humans"[mesh])
EMBASE	('PCNSL':ti OR ('diffuse large B-cell lymphoma*':ti AND ('central nervous system'/exp OR 'Central Nervous System':ti,ab OR 'CNS':ti,ab OR 'Cerebrospinal Axis':ti,ab OR 'Cerebrospinal Axes':ti,ab OR 'brain':ti,ab OR 'brains':ti,ab OR 'cerebral':ti,ab OR 'cerebro':ti,ab)) OR 'DLBCL':ti OR (('lymphoma'/mj OR 'Lymphoma':ti OR 'Lymphomas':ti OR Hodgkin*:ti) AND ('central nervous system'/mj OR 'Central Nervous System':ti OR 'CNS':ti OR 'Cerebrospinal Axis':ti OR 'Cerebrospinal Axes':ti OR 'brain':ti OR 'brains':ti OR 'cerebral':ti OR 'cerebro':ti OR 'intracranial*':ti))) AND ('cerebrospinal fluid'/exp OR 'Cerebrospinal Fluid':ti,ab OR 'Cerebrospinal Fluids':ti,ab OR 'Cerebro spinal Fluid':ti,ab OR 'Cerebro spinal Fluids':ti,ab OR 'CSF':ti OR 'myeloid differentiation factor 88'/de OR 'Myeloid Differentiation Factor 88':ti,ab OR 'MyD88':ti,ab OR 'CD79b':ti,ab OR 'CD79 antigen'/de OR 'CD79':ti,ab OR 'CXCL13 chemokine'/de OR 'CXCL13':ti,ab OR 'CXCL-13':ti,ab OR 'beta 2 microglobulin'/de OR 'beta-2 microglobulin':ti,ab OR 'beta2 microglobulin':ti,ab OR 'b2 microglobulin':ti,ab OR 'b2M':ti,ab OR 'neopterin'/de OR 'Neopterin':ti,ab OR 'neopterine':ti,ab OR 'drug therapy'/de OR 'drug therap*':ti,ab OR 'chemotherap*':ti,ab OR 'chemotherapy'/exp OR 'chemoradiotherap*':ti,ab OR 'antineoplastic agent'/exp OR 'methotrexate'/de OR 'methotrexate':ti,ab OR 'MTX':ti OR 'stem cell transplantation'/exp OR 'Stem Cell Transplantation*':ti,ab) NOT ('conference abstract'/it OR 'editorial'/it OR 'letter'/it OR 'note'/it) NOT (('animal experiment'/exp OR 'animal model'/exp OR 'nonhuman'/exp) NOT 'human'/exp)
