# Supplementary material for: The Diagnostic Yield of Cerebrospinal Fluid Analysis for the Diagnosis of Primary Central Nervous System Lymphoma: A Systematic Review
Source: Cancers (Basel). 2025 Jul 15;17(14):2352. doi: 10.3390/cancers17142352 (PMC12293505; doi:10.3390/cancers17142352)
Supplement: Supplementary file 1 [file cancers-17-02352-s001.zip › Table S2. Summary of risk of bias in the included studies..pdf]

Table S2. Summary of risk of bias in the included studies.

Study	Patient selection	Cytology	Flow cytometry	Reference standard	Flow and timing	Overall risk of bias
Feldheim (2024)	-	+	+	+	+	Low
Tatarczuch (2024)	-	-	-	-	-	High
Yi (2024)	+	-	*NA*	-	+	High
Janopaul-Naylor (2024)	-	-	*NA*	-	-	High
Batchelor (2024)	+	-	*NA*	-	-	High
Zhang (2024)	-	-	-	-	-	High
Schorb (2024)	+	-	*NA*	-	+	High
Chuang (2023)	-	-	*NA*	-	-	High
Bairey (2023)	+	-	*NA*	-	-	High
Ma (2023)	-	-	*NA*	-	-	High
Rozenblum (2023)	+	-	*NA*	+	+	Low
Zhong (2023)	+	-	*NA*	+	-	High
Li (2023)	-	*NA*	-	-	-	High
Lin (2023)	+	-	*NA*	-	+	High
Bazer (2023)	+	+	+	+	+	Low
Ebrahimi (2023)	-	-	*NA*	-	+	High
Wang (2023)	-	-	-	+	+	High
Das (2022)	-	-	-	+	-	High
Lage (2022)	-	-	*NA*	-	+	High
Ferreri (2022)	-	-	*NA*	-	+	High
Radhakrishnan Vs (2022)	+	+	*NA*	+	+	Low
Sang Eun Yoon (2022)	-	-	*NA*	+	+	High
Xiangwei Luo (2022)	+	-	*NA*	+	+	Low
Sun (2021)	+	-	-	-	+	High
Ji Yun Lee (2021)	-	-	*NA*	-	+	High
Yuki Yamagishi (2021)	-	-	*NA*	-	+	High
Mihir Gupta (2021)	+	+	+	+	+	Low
Ferreri (2021)	+	+	*NA*	+	+	Low
Seidel (2020)	-	-	*NA*	-	+	High
Shao (2020)	+	+	*NA*	+	+	Low
Houillier (2020)	+	+	+	+	+	Low
Sethi (2019)	+	-	*NA*	+	+	Low
Lin (2019)	+	+	*NA*	+	+	Low
Hiemcke-Jiwa (2019)	-	-	-	+	+	High
Rimelen (2019)	-	-	-	+	+	High
Mao (2019)	+	-	*NA*	+	-	High
Nayyar (2019)	-	+	*NA*	-	-	High
Patekar (2019)	+	-	*NA*	+	+	Low
Bromberg (2019)	+	-	*NA*	-	+	High

Mizutani (2018)	-	-	*NA*	-	-	High
Zorofchian (2018)	+		-	+	+	High
Ikeguchi (2018)	+	+	*NA*	+	+	Low
Hottenrott (2018)	-	-	-	+	-	High
Nam (2018)	-	-	*NA*	-	+	High
Ahn (2017)	+	-	*NA*	+	+	Low
Park (2017)	-	-	*NA*	+	+	High
Puligundla e al (2017)	-	-	*NA*	+	+	High
Jung (2017)	-	-	*NA*	+	+	High
Fan (2017)	-	-	*NA*	+	-	High
Cerqua (2016)	-	-	*NA*	-	-	High
Zhang (2016)	-	-	*NA*	-	-	High
Jang (2016)	-	-	*NA*	-	-	High
Liu (2015)	-	-	*NA*	-	-	High
Omuro (2015)	+	-	*NA*	+	+	Low
Pulczynski (2015)	-	-	*NA*	+	-	High
Sasagawa (2015)	-	-	*NA*	+	+	High
Liu (2015)	-	-	-	-	-	High
Olivier (2014)	-	-	*NA*	-	-	High
Ferreri (2014)	-	-	*NA*	+	+	High
Tao (2013)	-	-	*NA*	+	+	High
He (2023)	-	-	*NA*	-	-	High
Salamoon (2013)	-	-	*NA*	+	+	High
Rubenstein (2013)	-	-	*NA*	-	-	High
Korfel (2012)	+	+	*NA*	+	+	Low
Sasayama (2012)	-	-	*NA*	+	+	High
Wieduwilt (2012)	-	-	*NA*	-	-	High
Gerard (2011)	+	-	*NA*	+	+	Low
laack (2011)	-	-	*NA*	-	-	High
Omuro (2011)	+	-	*NA*	+	+	Low
Pasricha (2011)	-	-	*NA*	+	-	High
Ferreri (2011)	+	-	*NA*	+	-	High
Schoers (2010)	-	+	+	+	+	Low
Pels (2010)	-	-	*NA*	-	+	High
Hohaus (2009)	-	-	*NA*	-	-	High
Agarwal (2009)	-	-	*NA*	+	+	High
Ferreri (2009)	+	-	*NA*	-	-	High
Angelov (2009)	+	+	*NA*	-	-	High
Illerhaus (2009)	-	-	*NA*	+	-	High

Kiewe (2008)	+	+	*NA*	-	-	High
Haldorsen (2007)	-	-	*NA*	+	-	High
Yamanaka (2007)	+	+	*NA*	-	+	Low
Silvani (2007)	-	-	*NA*	+	+	High
Quek (2006)	-	-	*NA*	-	-	High
Kawamura (2006)	-	-	*NA*	-	+	High
Omuro (2005)	-	-	*NA*	-	-	High
Brevet (2005)	-	-	*NA*	+	-	High
Yamanaka (2005)	+	+	*NA*	+	+	Low
Hodson (2005)	-	-	*NA*	+	-	High
Korfel (2005)	+	+	*NA*	-	+	Low
Dubuisson (2004)	+	-	*NA*	-	-	High
Bessell (2004)	-	-	*NA*	+	-	High
Caroli (2004)	-	-	*NA*	+	-	High
Poortmans (2003)	+	-	*NA*	-	+	High
Abrey (2003)	-	-	*NA*	+	+	High
Ishikawa (2003)	+	+	*NA*	+	+	Low
Dabaja (2003)	-	-	*NA*	-	-	High
Choi (2003)	-	-	*NA*	-	-	High
Cheng (2003)	-	-	*NA*	+	+	High
Batchelor (2003)	-	-	*NA*	+	-	High
Braaten (2003)	-	-	*NA*	+	-	High
Ferreri (2003)	-	-	*NA*	+	+	High
DeAngelis (2002)	+	-	*NA*	-	+	High
Depil (2002)	-	-	*NA*	-	-	High
Calderoni (2002)	-	-	*NA*	-	-	High
Shibata (2002)	-	-	*NA*	+	-	High
Gleissner (2002)	-	-	*NA*	-	-	High
Goldkuhl (2002)	+	+	*NA*	+	+	Low
Herrlinger (2001)	+	-	*NA*	+	-	High
Mead (2000)	-	-	*NA*	+	-	High
Zylber-Katz (2000)	-	-	*NA*	-	-	High
O'Brien (2000)	-	+	*NA*	+	+	Low
Ng (2000)	-	-	*NA*	-	-	High
Wu (1999)	-	-	*NA*	-	-	High
Hiraga (1999)	-	-	*NA*	+	+	High
Guha-Thakurta (1999)	+	+	+	+	-	Low
Boiardi (1999)	-	+	*NA*	+	+	Low
Sandor (1998)	-	-	*NA*	-	+	High

Corry (1998)	-	-	*NA*	-	-	High
Cheng (1998)	-	-	*NA*	-	+	High
Brada (1998)	+	-	*NA*	+	+	Low
Blay (1998)	+	-	*NA*	+	-	High
Laperriere (1997)	-	-	*NA*	+	-	High
Glass (1996)	+	+	*NA*	+	-	Low
Schultz (1996)	-	-	*NA*	-	+	High
Schaller (1996)	-	+	*NA*	+	-	High
Krogh-Jensen (1995)	-	-	*NA*	+	-	High
Sarazin (1995)	-	-	*NA*	-	-	High
Blay (1995)	-	+	*NA*	+	+	Low
Grangier (NFT) (1994)	-	-	*NA*	+	-	High
Miller (1994)	-	-	*NA*	+	-	High
Selch (1994)	+	+	*NA*	+	+	Low
Glass (1994)	+	+	*NA*	+	+	Low
Hayakawa (1994)	-	-	*NA*	+	-	High
Liang (1993)	-	-	*NA*	+	+	High
Fusejima (1992)	-	-	*NA*	-	-	High
Remick (1990)	+	-	*NA*	+	+	Low
Michalski (1990)	+	-	*NA*	+	-	High
Brada (1990)	+	-	*NA*	+	-	High
Socie (1990)	-	-	*NA*	+	-	High
Grote (1989)	+	+	*NA*	-	+	Low
Pollack (1989)	-	+	*NA*	+	-	High
Vakili (1986)	-	-	*NA*	+	-	High
Bogdahn (1986)	+	+	*NA*	+	+	Low
Jellinger (1975)	-	-	*NA*	+	-	High

Abbreviations: - =High risk of bias; + = Low risk of bias
