# Supplementary figures and images for: The Diagnostic Yield of Cerebrospinal Fluid Analysis for the Diagnosis of Primary Central Nervous System Lymphoma: A Systematic Review
Source: Cancers (Basel). 2025 Jul 15;17(14):2352. doi: 10.3390/cancers17142352 (PMC12293505; doi:10.3390/cancers17142352)

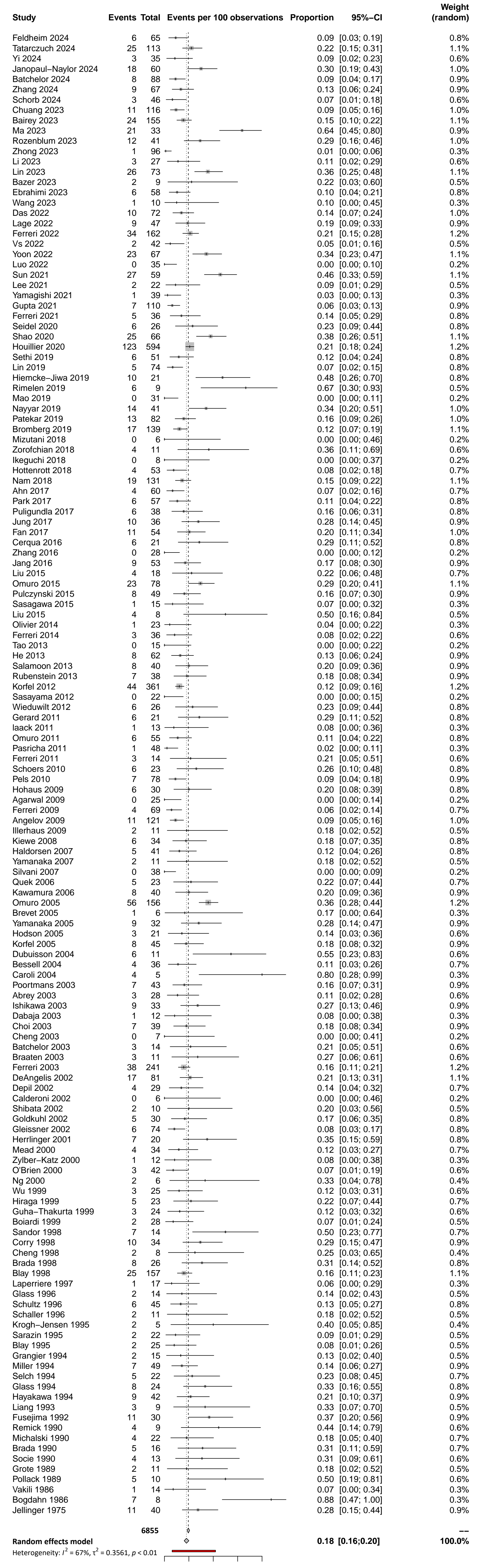

Figure S1. Forest plot of the detection rate of positive CSF results.

Supplement: Supplementary file 1 [file cancers-17-02352-s001.zip › Figure S1. Forest plot of the detection rate of positive CSF results..pdf]

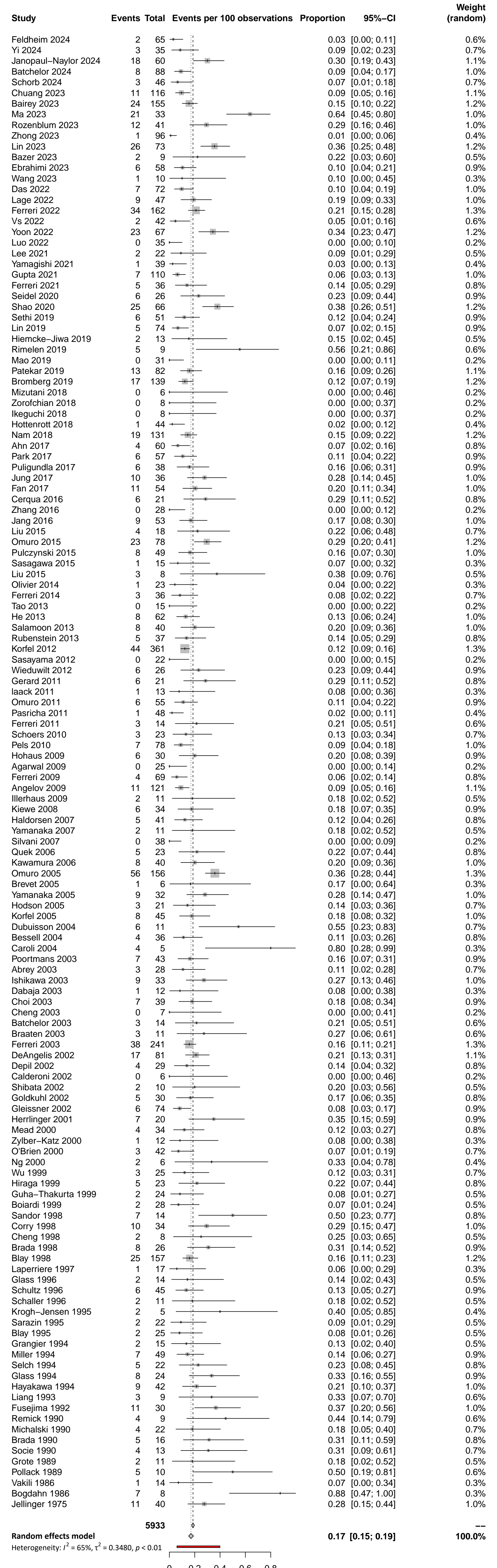

Figure S2. Forest plot of the detection rate of positive cytology results.

Supplement: Supplementary file 1 [file cancers-17-02352-s001.zip › Figure S2. Forest plot of the detection rate of positive cytology results..pdf]

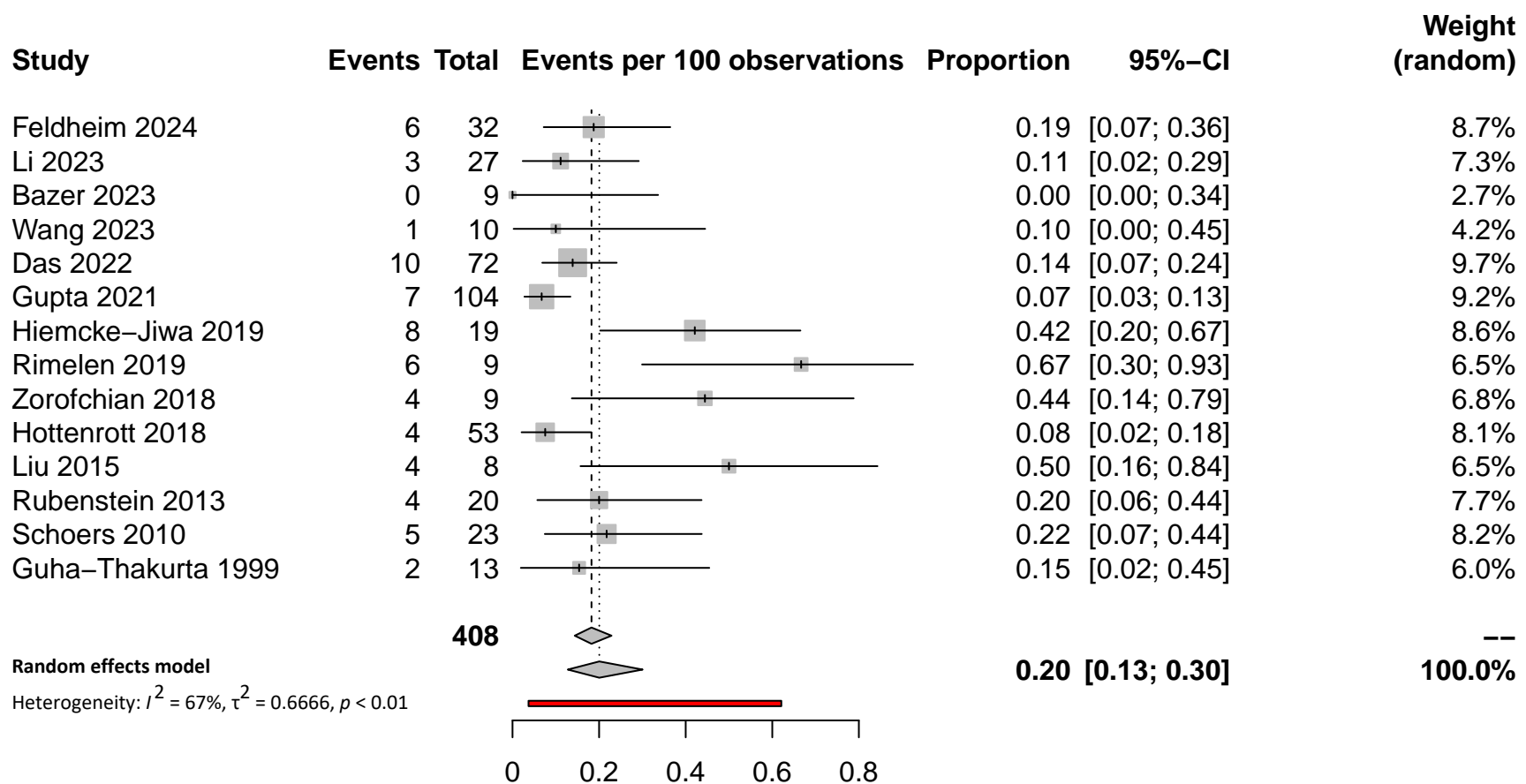

Figure S3. Forest plot of the detection rate of positive flow cytometry results.

Supplement: Supplementary file 1 [file cancers-17-02352-s001.zip › Figure S3. Forest plot of the detection rate of positive flow cytometry results..pdf]
